# A ‘civic turn’ in Scandinavian family migration policies? Comparing Denmark, Norway and Sweden

**DOI:** 10.1186/s40878-016-0046-7

**Published:** 2017-03-01

**Authors:** Emily Cochran Bech, Karin Borevi, Per Mouritsen

**Affiliations:** 1 Aarhus University, Aarhus, Denmark; 2 Södertörn University, Huddinge, Sweden

**Keywords:** Immigration, Family migration, Family reunification, Civic integration

## Abstract

Family migration policy, once basing citizens and resident foreigners’ possibilities to bring in foreign family members mainly on the right to family life, is increasingly a tool states use to limit immigration and to push newcomers to integrate into civic and economic life. The family migration policies of Denmark, Norway and Sweden range widely – from more minimal support and age requirements to high expectations of language skills, work records and even income levels. While in Denmark and increasingly in Norway growing sets of requirements have been justified on the need to protect the welfare state and a Nordic liberal way of life, in Sweden more minimal requirements have been introduced in the name of spurring immigrants’ labor market integration even as rights-based reasoning has continued to dominate. In all three countries, new restrictions have been introduced in the wake of the refugee crisis. These cases show how prioritizations of the right to family life vis-à-vis welfare-state sustainability have produced different rules for family entry, and how family migration policies are used to different extents to push civic integration of both new and already settled immigrants.

## Introduction

In Europe today, migrants’ rights to family life have come to depend on the country they move to. In Denmark, individuals with temporary asylum status must now wait three years for family reunification – a new restriction introduced in the wake of the European refugee crisis by the center-right government with support from most parties in Parliament – including both the populist Danish People’s Party and the Social Democrats. Widely condemned by jurists and NGOs as in possible breach of international law protecting the right to family life, this initiative is the last in a series of restrictions in this important, but less well researched area of immigration policy (Bonjour & Kraler, 2015; cf. Bailey & Boyle, 2004; Kofman, 2004). These include rules that have at times conditioned the right of would-be family migrants to join a spouse in Denmark on their own education and language skills, and have made Danish residents’ right to have spouses join them increasingly subject to *their own* current ‘integration standing,’ i.e. their employment and self-support record, and their language competence (Bech & Mouritsen, 2013). They have even included the possibility of evaluating children above age eight for their potential for ‘successful integration’. With these rules as with other immigration-related policies, Denmark has adopted a much stricter regime than neighboring Sweden (Borevi, 2015) and Norway (Siim & Skjeie, 2008; Staver, 2015), where family reunification of spouses, children and parents has been a great deal easier – at least until recently. In these countries too, economic self-sufficiency requirements and efforts to deter asylum seekers play a growing role. In the wake of the 2015 refugee crisis, Sweden – a state where the right-to-family-life perspective has otherwise remained particularly pervasive – in an effort to adjust regulations to a minimum EU level, has introduced a temporary law where asylum seekers who are not granted protection as Geneva Convention refugees in principle have no right to family reunification at all. Similar to the Danish reform mentioned above, this proposal has received devastating critique from human rights experts. Also, in the wake of the refugee crisis, the Norwegian parliament debated a proposal by the conservative government to delay family reunification for *all* categories of refugees, on top of already very restrictive requirements of financial self-support; the proposal was eventually watered down.

In all three countries, regulation of family reunification has been increasingly fitted into a broader ideological concern with what might be called welfare state civic universalism. By this we mean a set of normative and functional notions about how the welfare state should and can integrate newcomers in a way that promotes social welfare, gender equality and ‘thick’ ideals of individual autonomy, yet about how that welfare state is also fiscally and ‘culturally’ vulnerable. Interestingly, this otherwise shared Nordic conception of ‘the good life’ informs diverse, and even opposing, policy strategies of incorporation within the field of family migration – due to the differing priority given to human rights considerations. Within these strategies, the state may either function as a disciplining, socializing or supporting agent – by inducing economic self-support and labor market participation, by preventing forced marriages or by guarding the potential for welfare access of immigrants and citizens alike.

A more general trend towards civic integration requirements (Goodman, 2010, 2012a; Joppke, 2007a) is seen, in many immigrant-receiving societies, in citizenship acquisition rules, integration and anti-discrimination programs, education policies (Fernandez & Jensen, this volume) and labour market regulation (Breidahl, this volume). Western states, unfriendly to multiculturalism but wary of delegitimized, old-school assimilation tactics, try to mold the civic competencies, values and outlooks of newcomers – and often end up using heavy handed, even illiberal instruments and conditions (Joppke, 2005). Immigrants may express religious and cultural minority identities but must also become *good citizens*: they must be self-supporting, affirm liberal-democratic values, have good command of the host-society language and civic knowledge, and be loyal and inclined to participate in civic life.

The ideological rationale behind the ‘civic turn’ in its various versions (Mouritsen, 2008; 2013), informs not only integration and conditioning of access to permanent settlement and citizenship (with their associated rights), but also the regulation of *immigration* where states seek to select and screen not only work migrants, but even desirable refugees – and to ‘integrate from abroad’ family members who wish to join them. When in 2005 the Dutch parliament imposed a pre-entry integration regime on foreigners who wished to join a partner, parent or child in the Netherlands, requiring exams abroad to prove Dutch language ability and knowledge of society before admittance (Bonjour, 2014, p. 204; 2010), family reunification became a special arena of civic integration policies. It highlighted not only a more general linkage between immigrant *integration* and immigrant *selection*, but also a *double conditionality* imposed in this policy area – where both the civic deservingness of the sponsor and the civic integration potential of the incoming family member were evaluated. What was previously a basic human right to family life now had to be earned. And this logic is extending to a growing number of national settings.

In the Nordic type of comprehensive, generous welfare states, we would expect the tension inherent in family reunification policies between human rights perspectives and a sterner welfare state protectivist approach to be particularly salient, with the result that ‘civic selection’ ends up being more important than ‘civic integration’ – in the name of fiscal sustainability. The ambition to create new citizens is likely to be overshadowed by the call for sustained functioning of the welfare state for those who are already members. This approach is most evident in Denmark, but is also increasingly visible in Norway. In Sweden, there has been little civic conditioning in family migration policy, though this situation may currently be changing due to the large influx of asylum migrants (more than 160 000 asylum seekers during 2015). However, while recent proposals mean a significant restriction of Swedish family reunification policies, they do not include – so far at least – the kind of mandatory requirements and tests usually associated with civic integrationism.

This article discusses the development of family reunification since the late 1990s as a distinct area of migration and integration policy – focusing on the trajectories of the Nordic countries Denmark, Norway and Sweden. In doing so it demonstrates and discusses how the civic turn refers to conceptions of good citizenship that reflect the institutional and cultural reality of the welfare state, and the popular and political concerns mobilized to protect it – in Scandinavia, but probably also more broadly. Secondly, it argues that family reunification should be studied as a key field of civic integration in its own right, yet also serves as a lens through which to analyze Scandinavian civic integration philosophies in general. Thirdly, it demonstrates that these philosophies remain remarkably diverse, and in the case of Sweden and Denmark non-converging, even in this ‘most similar systems’ context – for reasons discussed elsewhere in this special issue (Borevi; Jensen & Mouritsen, this volume).

The article begins, in the section below, on a more conceptual note by challenging the meaning of the ‘civic’ in the literature on civic integration as being too narrow. This section is followed by a sustained comparative analysis of legislative changes in Denmark, Norway and Sweden, and the frames of argument used to justify them in public and political debate. In a concluding section, we then discuss how welfare-state uses of civic criteria to condition family migration rights depend on how access to the welfare state is conceived: where it is considered a universal right, rights-based logics prevail; where it is conditioned on deservingness (at least for foreigners), civic requirements (and civic selection criteria) outweigh rights-based perspectives.

## Family migration policy as a new domain for civic integration requirements

In response to real and perceived differences in socioeconomic outcomes and democratic commitment between immigrants and natives in many immigrant-receiving societies, policy-makers have from the early 2000s increasingly conditioned immigration, permanent residence status and citizenship on competencies and qualifications thought to make better outcomes more likely among immigrants. Yet these policies have varied greatly in the types and degrees of requirements introduced, to which phase of entry or status application they were applied, and the types of immigrants who were subjected to them. While the general trend was identified and theorized by a number of scholars (Green, 2007; Joppke, 2007a, 2007b), it was first more precisely operationalized by Sara Wallace Goodman (2010, 2012a). Goodman develops an index (CIVIX) to map out and systematically compare civic integration policies in 15 European countries. Index scores increase with the number and degree of requirements conditioning immigrants’ entry into a country, permanent residence, and acquisition of citizenship – requirements such as language course attendance or exams, civics exams or activity requirements, and fees (Goodman, 2014, p. 50).

However, while Goodman’s index helpfully structures comparison of how national policies are conditioning entry, permanent residence and naturalization on language and civic knowledge, it arguably misses key developments in civic integration regimes adopted in Scandinavia and other countries, some of them implemented through family reunification policy. First, one may problematize the *type* of requirements specified in Goodman’s operationalization of civic integration, which do not reflect the working definitions (Mouritsen, 2013) of what she herself calls “the attributes of membership,” understood as “civitas, the condition of citizenship” (Goodman, 2012b, p. 175). Goodman defines civic integration as the application of a particular set of requirements - including country knowledge, language, liberal values, integration courses, tests, contracts, interviews and oath ceremonies. Civicness here is understood as ‘republican’ (participation, loyalty) and ‘liberal-democratic’ (constitutional values).

This definition omits integration requirements revolving around economic criteria (other than fees for integration courses and tests). These include, for example, demands that immigrants must have achieved a certain employment record, be self-supporting, or have a certain income level or education. This ‘neo-liberal’ or social-democratic good citizen (e.g. Joppke, 2007b, p. 248) is a crucial component in policy changes in many family reunification regulations (as it is in changes to residence and naturalization rules). Another crucial aspect of recent family migration policy changes in many European countries is the introduction of stricter *age requirements* for spousal entry (higher than the legal marriage age for resident spouses) and for entry of children (in Denmark, low enough to allow integration), and blocks to those with *domestic violence records*. We argue that this type of requirement, though not tracked by Goodman’s civic integration definition (and index) is part of the larger civic integration trend in requiring newcomers to already have, or have potential to achieve, civic *maturity*. In Scandinavia this is understood to extend even to specific civic values and norms of family- and work-life related to gender equality and (women’s) individual autonomy.

Second, we contend that the set of rights (or legal statuses) being conditioned on civic integration requirements is actually broader than Goodman’s index allows, and that the scope of policies examined to compare such requirements such therefore be widened. Goodman specifies her index in terms of integration requirements for three ‘gates’: entry, settlement and citizenship. The policy subarea of family reunification revolves in particular around the first of these gates (entry), since it is concerned with regulating the *migration* of family members. And indeed, the CIVIX index does measure existence of pre-departure integration requirements, where civic conditions are targeted at family migrants (in the country of origin) to condition their possibilities to enter a country on the ground of family reunification. But the index does *not* take into account the implementation of civic requirements targeted at the *sponsor/reference-person* already resident in the country that condition foreign family members’ entry. Yet policy developments increasingly focus on the latter. Family migration policies differ from other migration policies because they concern not only, in the words of Laura Block, “outsiders knocking at a state’s doors and requesting entry” but also the “moral claims of insiders,” people living within state borders who ask to be united with their families (Block, 2012, p. 37; cf. Bonjour & Kraler 2015, p. 1412). Family reunification policies therefore form part of national ‘membership conditionality structures’ (Baldi & Goodman, 2015), often linked to the further ‘gates’ of permanent residence or citizenship, since the legal status of the resident *sponsor* required for family entry, such as permanent residence or citizenship, is dependent on meeting requirements conditioning that status. In sum, family reunification policies constitute one important part of the stratification of membership rights by civic integration requirements, and are affected by other policy areas concerning residence status and citizenship.

Finally, we contend that a comparative study should analyze cross-country variation also in the *argumentative structures* – the justifications, arguments and ideological rationales – that surround these policy measures. Civic integration marks a shift ‘from rights to duties’ – rights are used less to enable integration of individuals, and more to incentivize, positively or negatively, or force them be ‘good citizens.’ The way in which this rationale is adopted is likely to differ, however, depending on country-specific ideas about what good citizenship actually is, and about how societal integration and national cohesion comes about (Jensen, 2016). In the latter regard, Denmark and Sweden constitute each other’s opposites with the Danish society-centered bottom-up approach juxtaposed to the Swedish state-centered top-down approach (Borevi, 2017). Moreover, given the strong emphasis on the integration–immigration control nexus in family migration policies, arguments for human rights are likely to co-exist in tension with concerns to select immigrants who can contribute to the welfare state in the labour market and conform to the civic values and culture of the host society. The following analysis documents and compares not only what type of family migration policy instruments have been introduced – but also what arguments and justifications have been invoked to do so in each of the three countries.

## Divergent paths: Nordic states and the conditioning of family migration

In the late 1990s, the family migration policies of Denmark, Norway and Sweden were characterized by only slight differences, with all three countries prioritizing the right to family life over specific conditions. In the intervening years, however, the three countries’ rules in this area have diverged to become very different – displaying between them some of the most restrictive and liberal policies in Europe.

### Family migration policy in Denmark: sharp restrictions in the name of welfare

Denmark’s family reunification policies have been developed in successive policy changes since the late 1990s, with center-right governments introducing increasingly strict rules, and the center-left, in their periods of power, moving only to ease the most extreme restrictions but retaining the majority. The Danish rules have come to include requirements for age of spouses, attachment to country, language competence, self-support, employment records, and more, that each surpass similar rules in other countries, and together constitute the toughest family-migration rule package in force among Western democracies today.

Immigration to Denmark based on family reunification was available to spouses and children of all settled Danish residents without any significant exceptions until 2000, when the Social Democratic led government introduced a requirement that couples must have at least as strong a connection to Denmark as to any other country in order to be granted spousal immigration, as well as a requirement that a ‘suitable’ residence (of 20 m^2^ per person) was available. Through the late 1990s, Social Democratic mayors in the greater Copenhagen area had warned that national politicians, including the center-left, were out of touch with realities in residential areas dominated by immigrants. Per Madsen, mayor of Ishøj, said that “the country has to start all over every time an immigrant fetches a spouse from the home country. All over again with integration and all over again with children, who enter Danish kindergartens and schools without knowing the most minimal elements of the language” (cited in Hildebrandt, 1999). The mayors were concerned with numbers of arranged marriages and ‘ghettoisation’ in certain areas, and with easing the pressure on local welfare institutions (Christensen, 1999; Elkjær & Thomsen, 2001). Moreover, Herlev mayor Kjeld Hansen said it made “no sense to invite people to Denmark, if we do not have a real integration possibility to offer them. Hence we must establish whether the applicant in terms of language, culture and values is capable of adjusting to the Danish labour market” (cited in Langager & Maressa, 2001).

The Social Democrats’ rule changes in 2000 paled, however, in comparison to the restrictions introduced by the succeeding Liberal-Conservative government (2001–11). Citing a primary goal of reducing the number of family migrants and additional aims of 1) reducing forced marriages of immigrant-background young people living in Denmark to partners from their families’ home countries, and 2) reducing the number of family migrants on public welfare, that government introduced some of the strictest family migration policies in Europe. Shortly after gaining power, with parliamentary support from the Danish People’s Party, it introduced a ’24-year rule’ requiring that both partners be at least 24 years old to be granted spousal entry, and stiffened the attachment requirement to demand that couples have a *greater* attachment to Denmark than to another country. In 2004 this latter rule was made only to apply to those couples whose resident partner had not been a Danish citizen for 28 years or more – therefore to all young people under 28 and most immigrants. Additionally, the resident spouse was required to prove they could support the incoming spouse with means to live on and to put up a bank guarantee of 50,000 DKK as a caution in the case of any support received from the public sector.

Over the following eight years, that government increased the attachment requirement to demand ‘a *much greater* attachment to Denmark, and introduced a ‘point system’ whereby incoming partners must qualify for entry with a combination of qualifications including longer educations, job experience, and competence in the Nordic languages, English, French, German or Spanish. Partners under 24 could enter provided they scored extremely high on this scale (The Danish Government and Danish People’s Party, 2010). They also required that the resident partner pass an intermediate Danish language exam (*Danskprøve 2*, European level B1/B2), be actively employed, and not having received public assistance in the previous three years. The required bank guarantee was increased to 100 000 DKK. These requirements increasingly subjugated Danish residents’ right to family life to their own and their incoming partners’ potential for contributing actively to the Danish tax base (rather than being welfare recipients) (Bech & Mouritsen, 2013).

These issues remained intensely politicized and concerns with legality, equal treatment and human rights became increasingly marginalized over that decade. While in 2001 even the Liberal Party pledged respect for international conventions (Boddum, 2001), and the Social Democrats (after criticism in 2004 from the Council of Europe and the UN) joined the small Social Liberal party in 2004 in an unsuccessful bid to establish a family reunification complaints board, by the end of the period only the Social Liberals and the far left had such concerns. Instead, issues of gender, authoritarian families and protection of young people against forced marriages dominated the agenda, as in Minister for Integration Bertel Haarder’s typical invocation of “a devastated girl, crying at the counter at the Immigration Agency begging for a rejection [of an application], or the girl who, having given up all hope for help from the Danish society, merely asks the authorities to lengthen the case handling” (Haarder, 2003). Politicians, also Social Democrats, discussed whether the 24-rule “worked”, both to “regain control of immigration” and “break the tradition” of arranged marriages so that “more young independent immigrant women …[might] create their own lives” (Integration Minister Rikke Hvilsø, cited in Svane & Borg, 2006; Fortsat få familiesammenføringer [Still few family reunifications], 2006).

Concerns over numbers of family migrants remained pronounced, with politicians expressing dismay at the “damage done” by EU legislation and European Court of Human Rights judgments (such as the 2008 Metock ruling), fears that the 24-year rule merely delayed family reunification, and reassurance that the higher numbers reflected imported spouses from Thailand and the Philippines, rather than Turkey (Henriksen, 2008; Steensbeck & Flores, 2008). Throughout the period, however, there remained some controversy over the fact that tightened rules affected ‘real’ Danes, who found themselves unable to bring their (mostly Western) husbands and wives to Denmark – something that the business community in particular criticized as an unfortunate negative branding of the country.

Following the financial crisis, discourse on family reunification increasingly linked civic integration to economic growth agendas. Presenting the early 2010 *New Times, New Demands* policy package that introduced the above-mentioned point system and assigned negative scores for living in one of 42 residential areas designated ‘ghettos,’ Liberal Integration Minister Søren Pind emphasized that “Denmark wants to attract foreigners who can demonstrate civic values and create growth and prosperity” (Minister for Refugees & Immigrants and Integration (S. Pind), 2011). His predecessor Birthe Rønn Hornbech had similarly stressed that the government “wants you to have some qualifications in order to immigrate on family grounds” (cited in Nielsen, 2010). In an effort to project a new center-left hard line, the Social Democrats and Socialists proposed their own, less restrictive point system, which contrasted a more voluntaristic will-to-integrate logic with the government’s neo-liberal discourse, asking, “Should people who wish to migrate under family reunification to Denmark be evaluated on their contribution to the gross national product? Or … on their wish and will to become part of Danish Society?” (Kristensen & Krag, 2010). But they did not object to integration demands of language skills on family-reunified spouses per se, as that would help immigrants by preparing them for education or the labour market – helping them avoid a fate of “standing without competences, hidden away in a ghetto” (Kristensen & Krag, 2010).

In addition to raising requirements for spousal reunification, the liberal-conservative government also introduced rules to make it more difficult for some foreign citizen residents to bring their children to join them. In 2004, lawmakers introduced new rules to restrict child entry to those under 15 years of age unless quite special circumstances speak for it. This move was justified by the government as making it harder for immigrants’ children to be sent on longer ‘re-training’ stays (in Danish *genopdragelsesrejser*) in their parents’ home country, seen to be used by conservative immigrant communities to discipline young people into a closer cultural and religious connection to the home country rather than a Danish way of life. However, the government noted that it would use the available exception to allow the entry of teenage children in cases where it would help meet Denmark’s need for labor in particular sectors such as engineering, natural sciences, technology and the medical professions (Committee for Immigration and Integration Policy, cited in Minister for Refugees & Immigrants and Integration, 2004). The opposition voiced criticism; the Social Democrats insisted that a child’s welfare should be the primary consideration also in cases involving 15–17 year-olds, and the Social Liberals objected that the law would not prevent many cases of ‘re-training,’ but instead effectively prevent children aged 15–17 from joining their parents in Denmark.

Another change introduced in the same law made bringing in some children conditional on their having “potential for successful integration” – in cases where one parent still lived in the child’s home country and two years had passed beyond the resident parent’s initial qualification (Law 171, 2004). Such potential was to be judged on the length and character of the child’s residence in Denmark and their home country, whether the child spoke Danish and the home-country language, and whether the child’s experiences “had been influenced by Danish values and norms to such a degree that the child had or had potential to gain an attachment to Denmark to enable successful integration” in Denmark (Law 171, 2004). In addition, this was also to be evaluated by examining “whether the resident parent was well-integrated and had a strong attachment to the Danish society” (Law 171, 2004).

After 10 years of steepening restrictions, the Social Democratic-led, center-left government in power 2011–15 eased the sharpest rules. Most significantly, they removed the competence-based point system for entering spouses, removed application fees, and decreased the required language competence for resident partners to lower-intermediate (*Danskprøve 1,* A2/B1). They also changed the attachment requirement back to the former (2002) language of ‘greater attachment’ to Denmark, made that requirement apply to spouses of those who had been citizens for 26 rather than 28 years, and returned the bank guarantee requirement to 50 000 DKK. The 24-year rule remained unchanged, as did the requirements for self-support, employment record and housing. Relating to entry of children, in 2014 the center-left government adjusted the requirement for integration potential to be applied to children age eight or above, rather than all (as before, in cases when a resident parent had qualified for family entry for more than two years and when another parent remains in the home country). Taken together, the center-left government kept most of the previous government’s restrictions at levels on or about what they were in 2009. A minority Liberal government took power after the June 2015 election, with parliamentary support from the Danish People’s Party, the Liberal Alliance Party and the Conservatives. At the time of this writing, that government has made a several changes to family migration policy, and plans more. It has re-introduced an application fee of 6000 DKK (€805) and the higher bank guarantee requirement for spousal entry. It has also once again increased requirements for permanent residence – which affect when resident foreigners qualify to bring in family members – by requiring a higher language exam (B1/B2 level), and a full-time work record, and more years of residence (8 for permanent status, and thus 11 for family entry - though fulfillment of extra requirements allow acquisition in 6 years; The Danish Government/Regeringen, 2016).

At this time the Social Democrats tightened their own demands for family reunification, expecting this “both to have a positive effect on numbers … and on integration, because those who come will be more suitable for integration” (SD integration spokesman Dan Jørgensen, cited in Kristensen, 2015). The Social Democrats and the Danish People’s Party emphasized welfare over humanitarianism when they agreed not to spend funds to shorten case processing time for family reunifications, as this would cause local councils to have less money to spend on daycare and hospitals (“S og DF,” 2016).

At the same time, two 2016 European court decisions ruled two of the Danish rules discriminatory – the rule requiring that any child over age eight with one parent in the home country and whose Danish-resident parent had been eligible to bring family in for *more than two years* must be subjected to a ‘potential for integration’ evaluation (European Court of Justice, Genc C-561/14); and the rule that the attachment requirement *only be applied to couples where one spouse had been a citizen for 26 or more years* (European Court of Human Rights, Biao v. Denmark) – this second decision on the grounds that it constituted ethnic discrimination. The Danish government respondent to these rulings by dropping those distinctions, and applying the rules more broadly – to all children eight or older with a parent in the home country, and to all couples who apply for spousal reunification (Immigration & Denmark/Udlændingestyrelsen, 2016a, 2016b).

In addition, after the flow of refugees increased sharply in 2015, the Liberal government and its support parties as well as the Social Democrats rushed passage in January 2016 of a law that tightened access to family reunification for refugees and others who came as asylum-seekers. While that legislation was widely criticized for its provision to allow police to confiscate asylum-seekers valuables over a certain value (the ‘jewelry law’), its lesser-known clauses changed access to family reunification by requiring refugees to pay for the costs of transporting their families to Denmark, previously paid by the Danish state, at their own expense; and required those with temporary protected status (not convention refugees) to wait three years before applying to bring their families to Denmark. Leading voices from other opposition parties, human rights scholars and refugee-helping organizations protested the three-year waiting period, citing human rights law and the possibility that waiting children’s development could be damaged and that family members could experience great deprivation or even be killed while they waited near or in conflict zones (Drachmann, 2016; Danish Institute for Human Rights, 2016, p. 2). Immigration Minister Inger Støjberg was aware of the potential problems, saying, “We have gone to the edge of the conventions, and it is correct that there is a procedural risk in relation to the question about family reunifications. But that is a risk I am willing to take” (cited in "Støjberg tager risiko", 2016). The government did not defend the rule change on its own merits, but promoted as a package of restrictions on asylum-seekers and refugees that were meant to make Denmark less attractive to asylum-seekers, the ultimate goal being to protect the state of Danish social cohesion and the welfare regime. “While we wait for an international solution,” said Prime Minister Lars Løkke Rasmussen in relation to the new rules, “it is our job to protect Denmark” (Jørgenssen, 2015). Requirements for family reunification to Denmark are summarized in Table 1.

**Table 1:** Requirements for family reunification to Denmark

Requirement type	On foreign-citizen resident reference person	On entering spouse	On entering child
Language, Knowledge	Pass language exam (B1/B2 or equiv.)	At entry: Pass language exam within 6 months of entry (A1) After entry: Participate in obligatory integration program	-- (Though see below)
Economic resources, stability, self-support	Bank guarantee: 100 000 DKK (for spousal entry) Adequate housing (20+ m2/ person) Record of self-support Record of employment	Fee: 6000 DKK (€805) (does not apply to family applying to join refugees)	--
Age limit	Over 24 years (for spousal entry)	Over 24 years	Under 15 years
Domestic violence record	Must show none	Must show none	
Length of residence	11+ years if not entered through asylum (perm. res. for 3+; may be 9 total if achieved perm. res. using extra qual. introduced in 2016); if refugee, 1 year; if temporary protected status, 3 years	--	--
Attachment to country	Greater than to any other country (as couple, for spousal entry)		Children 8 or older with one parent still in country of origin must be evaluated to have ‘potential for successful integration.’

### Family migration policy in Norway: on your own dime

In Norway, family migration polices have been less demanding and complex than the Danish rules, yet require more than the Swedish. Family reunification is not conditioned on educational or language requirements for resident or entering family members, but rather on the resident family being self-supporting and having certain income levels. In the wake of the refugee crisis, and at the initiative of Integration Minister Sylvi Listhaug, of Norway’s new-right Progress Party, conditions and waiting periods have become significantly tightened.

Leading up to and during the early 2000s, foreign residents in Norway were generally able to bring in spouses and minor children with few limitations other than basic requirements for adequate housing. In 2004, the Immigration Act Commission (IAC) published a proposal for possible reforms, initiating political discussions that culminated in new rules for family migration introduced in 2007 and 2010 – all under the long Social Democratic government led by Jens Stoltenberg (2005–13). The commission raised the problem of forced marriages between young people in Norway of immigrant descent and new foreign spouses, and proposed a requirement that both spouses be at least 21, to ensure they were mature enough to resist family and cultural pressure (Immigration Act Commission, 2004, p. 243). While the commission noted that liberal family reunification policies worked to attract asylum seekers to the country, it did not deem using family migration rules to deter asylum seekers appropriate because they “risk serious abuses in their home country, and there is no other way to ensure family unity than through family reunification in the country of refuge. Respect for family unity in cases where one of the family members are entitled to stay in Norway in accordance with our international obligations, must be an obvious precondition for Norwegian family immigration” (Immigration Act Commission, 2004, p. 214).

While the Labour Party promoted the proposal to introduce an age requirement, the other two coalition parties were more hesitant (Siim & Skjeie, 2008). Significant criticism of the proposal was voiced at a public hearing in 2006, highlighting that the requirement would also affect persons who marry voluntarily, that it might cause young people to be sent to their family’s home country to marry and remain there (Labor and Inclusion Agency 2007, 9.6.3.8; Skjeie & Teigen, 2007). The government concluded in the 2007 bill on immigration that “even though the practice of arranged marriage can be seen to challenge ideals of freedom and equality in Norwegian society, that is not a basis for setting restrictions on access to establishing a life together as partners themselves wish” (Labor and Inclusion Agency, 2007, 9.6.3.9).

In 2008, a law change was introduced to require self-support of all resident partners requesting entry for existing or new spouses, including Norwegian citizens above 23 years (who had previously been exempted). The requirement was justified as a way to reduce welfare dependency and to fight forced marriages, reasoning that it would promote economic integration by incentivizing young immigrants to become self-supporting, and further that a self-supporting person would be better equipped to resist family pressure about whom to marry (Eggebø, 2010, p. 302–3; Jordheim, 2008). As Helga Eggebø notes, it is ironic that while the new income requirement was intended to incentivize more resident spouses to be independent of their families’ support, the same rule - by not including incoming spouses’ income in the tally of the required amount – assumed the latter to be dependent on their already resident partners (Eggebø, 2010, p. 304). At the same time, she notes, the new rules further amplified this potential dependence by raising from three to five years the time an imported spouse must remain in a marriage to retain the residence permit (Eggebø, 2013).

In 2009, a requirement was introduced that persons allowed to reside in Norway on humanitarian grounds must have worked or studied four years in Norway before being allowed to bring in any new family members (Norwegian Parliament, 2009). In 2010, the income requirement was further raised to a level above the Norwegian minimum wage (NKR 242.440/EURO 25.586 per year). Further raises were contemplated, though not to the level proposed by the present right-wing government led by Erna Solberg (NKR 304.500/EURO 32.135), which also proposed to copy the Danish 24-year rule (the age proposal was not adopted at this time, but was in 2016).

While openly inspired by Denmark, Norway’s policies in the area of family reunification, with the exception of the high income requirement (one of the highest in Europe), are still gentler. An obligatory introductory program (language and social orientation) for family members applies once they have been granted admission to Norway, and participation conditions social benefits as well as settlement and citizenship rights, but there are no tests. Also, no conditions apply for reunification with children under 18 years of age. The law states that, when both parents have residence, an applicant under 18 “have the right to a residence permit;” and the same where only one of the parents has, unless the child’s interest weighs against it (Utlendingsloven, § 42). Refugees (but not those with humanitarian permits) who apply for reunification (not family *formation*) are exempted from the income requirement during their first year in Norway, to ensure family unity.

Compared to Denmark, the Norwegian debate has emphasized self-support – reflecting the importance of universal labor market participation (*‘arbeitslinien’*) more than age or maturity as a key goal in public policy, although the latter concern is evident in the discourse (Brochmann & Hagelund, 2012). There has also been slightly more political consensus on this issue in Norway than in Denmark, and newspaper coverage has been less extensive, at least until recently. In addition, Norwegian politicians did not openly cite the reduction of numbers as a separate goal of policy, as their Danish counterparts did, and Norway’s humanitarian concern with the right to family life of refugees is constantly noted.

With the refugee crisis and mounting concern over the sustainability of social cohesion and the welfare state in a time of falling oil prices, this is beginning to change. In November 2015, in the midst of a highly politicized concern with the refugee influx, even regarding the Northern border to Russia, the Conservative/Progress Party government laid out a 15-point list of stricter measures, some of which were not spelled out in detail (Glomnes, 2015). This found some agreement even from the Labour Party as well as the small Social Liberal and Christian parties, all of which had previously moved to try to contain the anti-immigration Progress Party. After extensive consultation, a softer 18-point framework agreement (between all parties except the Socialists, the Greens and the small Agrarian Center Party), was presented. This met the need “for people within and outside Norway to know that we are in control of our borders,” according to Labour Party Leader Jonas Gahr Støre (cited in Kristiansen, Ruud, & Glomnes, 2015). But it was also hailed on the Progress Party’s website as bringing “Europe’s toughest asylum policies” (Fremskrittspartiet, 2015).

The final government proposal presented in April 2016 included a new requirement that immigrants and *all* refugees must have had three years of work or education to be eligible for family reunification; that reunification may be denied those with temporary status “if their family life may be led safely in a third country, to which the family as a whole has a stronger attachment;” a requirement that reference persons must support their family members “from day one;” and an introduction of a 24-year rule resembling the Danish one, though with more room for dispensations (Gjerde, 2016). The government proposal was fiercely criticized by human rights organizations and even leading personnel in government offices (e.g. Frode Forfang, head of UDI, the Norwegian Foreigners’ Directorate; and IMDI, the Norwegian Directorate for Integration and Pluralism; see Tjernshaugen, 2016). The Labour Party (which had been sitting on the fence for some time) and the smaller center parties were opposed to the three-year work or education requirement (Amundsen, Skarvøy, & Johnsen, 2016). In the end this requirement was rejected, while the attachment criterion and the 24-year-rule went through. The parliament also commissioned the government to prepare a law change to include a “quarantine” principle for family formation: before being allowed to bring in a *new* spouse or partner from abroad, the reference person must first have had six years of work or education in Norway.

Despite this tightening, it may be argued that political justifications given for Norway’s rule changes retain a different normative flavor from those in Denmark: while the civic integration agenda in Danish family reunification policy is increasingly dominated by talk of reducing numbers of incoming family migrants and of undesirable categories of immigrants, they remain somewhat less articulated in the Norwegian debate, outside the ranks of the Progress Party.

This reflects a strong humanitarian element in Norwegian national self-conceptions that places the country closer to Sweden than to Denmark. Even Conservative Prime Minister Erna Solberg estimated in an April 2016 interview that Norway could manage a yearly intake of 30 000 refugees (slightly higher than in 2015); she also addressed the challenges of labour market integration and the duty of ordinary Norwegians to be more open and welcoming (Johannessen, 2016). In a similar vein, Labour Party immigration spokesperson Helga Petersen stressed in November 2015 “our possibilities to take responsibility for our small share of the world’s refugees,” citing the international conventions’ protection of refugees’ right to family life (cited in Bugge, 2015). Most significantly, all parties continue to link talk of limiting the number of incoming migrants and refugees to the capacity for civic integration (and avoiding radicalization), and to the potential for reunited family members to ensure newcomers’ integration into labour markets. Tougher self-support requirements are expected to incentivize applicants, in the words of the Progress Party’s Minister for Integration Sylvi Listhaug, to “make the effort necessary to integrate” (cited in Gjerde, 2016). Requirements for family reunification to Norway are summarized in Table 2.

**Table 2:** Requirements for family reunification to Norway (2016 amendments in italics)

Requirement type	On foreign-citizen resident reference person	On entering spouse	On entering child
Language, knowledge	--	After entry: participate in introductory program (no test)	--
Economic resources, stability, self-support	Adequate housing Income min. €29,500/ year No social assistance in past 12 months 4 years full-time employment or study in Norway – for family formation of indivs. with asylum or protected status *2016 proposal: 3 years full-time employment or study in Norway – for all family reunification, to apply to all individuals with asylum or protected status*		--
Age limit	*2016 proposal:* *Over 24 years (for family formation, spousal entry only)*	*2016 proposal:* *Over 24 years (family formation)*	--
Domestic violence record	Must show none	Must show none	
Length of residence	-- (perm. residence or resident permit that can lead to perm. residence)	--	--
Attachment to country	*2016 proposal: Greater than to any other country (as couple, for spousal entry)*		--

### Family migration policy in Sweden: minimal dents in the rights framework

Swedish family reunification policies remain among the most liberal in Europe. Also in a Nordic comparison, Sweden is clearly positioned at the liberal end of the spectrum, most notably juxtaposed to Denmark. Consequently, and in sharp contrast with the multitude of Danish policy changes, Swedish reforms in this policy area have been few. Up to 2016, only two restrictive changes were made: the first in 1997, when possibilities to bring in family members outside of the so-called nuclear family were limited, and the second in 2010, when a financial support requirement was introduced. In comparison with recent policy changes across Europe these are rather minor restrictions, both in scope and content. A new policy initiative, launched in November 2015 and implemented in July 2016, will however mean a drastic cut in family reunification rights also in Sweden, at least temporarily.

In Sweden entering family members are not required—either prior or after entry—to prove their *civic deservingness* via any courses, language tests, etc., and there is no corresponding demands on the sponsor to condition family reunification. Similarly, the idea of introducing specific *age requirements* for transnational spousal migration has not received much political support in the Swedish context. Such requirements have been seen to run counter to the Swedish immigrant integration and welfare state ideology to treat immigrants and natives on equal terms (cf Borevi, 2014). For example, in 2012 a Swedish government appointed inquiry into forced marriages and child marriages rejected the idea of introducing higher minimum marriage age for transnational marriages, “since it would mean a strong limitation of an adult persons’ right to marry and live together and a negative special treatment of transnational marriages” (SOU 2012:35, p. 400).

Also in relation to *economic requirements* to condition family reunification, Sweden has taken a notably liberal position: though such requirements have existed since 2010, they are low by European standards and, more crucially, require only self-support of the sponsor, but *not* the ability to economically maintain an incoming relative. While the latter idea was before the 2010 reform, this was only in relation to entry of relatives beyond the ‘nuclear’ family and as a way to maintain a more liberal approach. In 1997 a Social Democratic government carried through restrictions for this category of family migrants in 1997, with support from the Centre Party and the Conservative Party; all other parties in parliament voted against. The measure was justified as giving needed cuts in welfare state expenses. Up to then, the Swedish rules had been notably liberal, normally allowing family reunification to parents and grandparents older than 60 years as well as adult and unmarried children, if it could be demonstrated that in some essential aspect the relative was *dependent* on the family member who had moved to Sweden or was the “final link” whose entire network of close relatives had already migrated to Sweden.

In the committee report preceding the 1997 government proposal, a financial support requirement for relatives beyond the nuclear family had been brought up as a possibility to keep more generous admission rules for family members beyond the nuclear family, but was in the end rejected by the majority on the grounds that it “would not correspond to the prevailing principles in Swedish society in general” and was seen to be incompatible with both the Swedish universal welfare state system and the policy goal of treating immigrants on the same terms as natives (SOU 1995:75, p. 164), an opinion which was embraced also in the subsequent government bill (Government bill, 1996/97 (1997):25, p. 115). In the following years, the idea surfaced a couple of times; in 2002 a committee majority wanted to introduce a support demand for relatives beyond the nuclear family and also for new spouses – the latter justified as a way to help protect young people – particularly women – from being persuaded or forced to enter a marriage against their will (SOU 2002:13), but these proposals were criticized as running counter to the principle of Swedish welfare state universalism (SOU 2005:103, p. 116) and never materialized.

As mentioned, in 2010 a self-support requirement for entry of nuclear family members was eventually introduced by the right-of-center Alliance government (2006–2014). Unlike previous proposals, this reform was neither justified nor designed to guarantee that sponsors would take economic responsibility for their family members. In fact, no such demands were made – the requirement was only that the sponsor must be able to earn his or her *own* living. This construction situated the policy as less stringent than allowed under the 2003 EU Family Reunification Directive (Council, 2003/86/EC) where member states are allowed to require sponsors to have “stable and regular resources which are sufficient to maintain himself/herself *and* the members of his/her family, without recourse to the social assistance system of the Member State concerned” (emphasis added). In terms of *housing*, however, the sponsor was required to have suitable accommodation for the entire family.

The 2010 requirement was justified primarily as a way to incentivize new arrivals to integrate; to “promote integration by increasing incentives for people to obtain work, earn their own living and move to municipalities where they have a good chance of obtaining work and a place of their own to live” (Government bill, 2009/10:77 (2009); SOU 2008:114). The opposition parties – the Social Democratic Party, the Left Party, and the Green Party – were profoundly critical to this approach, arguing that the possibility to reunite with one’s close family must be seen as an indispensable precondition for people to successfully integrate, not as a reward. The support requirement was also criticized for representing a prejudiced and faulty assumption that newcomers would not make an effort to find a job or a place to live unless there was an explicit requirement to do so (Parliamentary Committee on Social Insurance, 2009/10:16 (2009)). But the issue also provoked substantial disagreements within the four-party coalition government. The Conservative Party was the driving force in favor of a requirement, whereas the Christian Democrats were profoundly critical to any limitations on the possibilities to reunite with the close family. The Liberals and the Centre Party took something of a middle position, supporting the general idea of requiring new arrivals to get a job before bringing in the family, but simultaneously anxious to secure exemptions, for example, for unaccompanied minors and refugees. These disagreements resulted in a government proposal that was significantly watered down (as compared to the proposals of the government-appointed inquiry in 2008, see Government bill, 2009/10:77 (2009)), including an extensive list of exemptions that in practice meant that extremely few people were submitted to the new support requirement. The policy was approved by the parliament in March 2010. Of all family reunification cases handled by the Migration Board in the years after the introduction of the support requirement, less than 1% were actually subject to it.

After the elections in September 2010, the Sweden Democrats entered parliament. This party has gone against the grain by campaigning for a policy similar to the Danish, including a ‘24 years rule’ for transnational marriages; a ‘national attachment requirement’ and an obligation for both spouses to sign a declaration pledging themselves to take part in social life and comply with Swedish laws plus a range of economic requirements. The Sweden Democrats have also echoed arguments dominant in the Danish context, presenting restrictions as necessary to protect national identity, to limit the state’s immigration-related expenditures, and to reduce men’s violence against women (e.g. Parliamentary Committee on Social Insurance, 2011/12:12 (2012)).

During the fall of 2015 a number of political initiatives were taken in response to the all-time high refugee migration. On 23 October, all parliamentary parties except the Left Party and the Sweden Democrats (the former decided not to take part in the agreement and the latter was not invited) reached an agreement including the decision to only admit time-limited permits to asylum seekers – presented as a temporary deviation from the principle to grant immediate security of residence (Överenskommelse, 2015). Quota refugees, unaccompanied minors and families with children were, however, exempted from the agreed restriction (so could still receive permanent residence), and it therefore amounted to quite small changes in terms of family reunification rights (though the self-support requirement was expanded to apply to family formation cases involving Swedish citizens, EU citizens and people with more than four years of residence).

As asylum seekers kept arriving in record-high numbers, the sense of crisis grew in importance, pressing the government to find solutions. On 24 November, only a month after the six-party-agreement, the Social Democratic and Green Party coalition government announced a number of additional actions intended to limit the refugee flows (Government Offices of Sweden, 2015). One measure was to temporarily introduce ID checks at Sweden’s borders – these took effect on 4 January 2016. Further, the government explained that a temporary adjustment of the Swedish asylum policies to the “EU minimum” was needed in order “to limit the refugee flow and put pressure on other EU countries to take their share of the refugee burden.” This meant that most asylum seekers would only achieve time-limited residence: 3 years for Geneva Convention refugees, and 1 year for persons with alternative protection status (quota refugees would, however, continue to achieve permanent residence). Unlike the six-party-agreement, this initiative had drastic consequences for family reunification. Formulated to meet no more than the minimum requirements (as stipulated in the EU directive on family reunification), the right to rejoin with the close family was only to be granted to convention refugees (provided the application was turned in within 3 months after the asylum decision), and hence denied to those with alternative protection needs. Similarly, following the ambition to temporarily bring Swedish regulations in line with the more restrictive policies found in other EU countries, a reference person must not only be self-supporting, but also be able to financially maintain the incoming family member. Quota refugees and children were exempted from the support requirement, as were asylum seekers who had turned in their application prior to 24 November 2015, when the announcement of changes had been made. The proposal also involved an increase in the required minimum age for spousal reunification, from 18 to 21 years, but this was justified solely as a way to adjust Swedish regulations to the less liberal “EU minimum” (and is applicable only if the reference person is granted asylum under the new temporary law; Government bill, 2015/16:174 (2016)). Arguments about “civic maturity” or prevention of forced marriages have been conspicuous by their absence.

The proposal received massive criticism from various consultation bodies, who argued that it ran counter to human rights concerns and would lead to contra-productive results in terms of integration. The proposed restrictions in asylum rights – and particularly the cuts in family reunification – also provoked heated internal conflict and criticism within the governing coalition, particularly in the refugee-friendly profiled Green Party, which experienced a dramatic fall in popular support and a replacement of one of its two main leaders as a result. But the issue also divided the right-of-center parties: while the Center Party, the Christian Democrats and the Liberal party deplored that the protection of reunification rights for families with children was now abolished, the Moderate party welcomed the stricter rules and thought the government should have gone even further. Similarly, the Sweden Democrats criticized the proposal as “too little, too late.” It was rejected altogether by the Left party (Parliamentary Committee on Social Insurance, 2015/16:16 (2016)). The proposal was accepted in parliament on 21 June and took effect a month later, on 20 July 2016. Requirements for family reunification to Sweden are summarized in Table 3.

**Table 3:** Requirements for family migration to Sweden (2016 amendments in italics)

Requirement type	On foreign-citizen resident reference person	On entering spouse	On entering child
Language, knowledge	--	--	--
Economic resources, stability, self-support	Have adequate housing Self-support (via employment; entrepreneurship or work-related benefits) But many exemptions *Temp. law (2016–2018): Most exemptions removed; requirement extended to cover also ability to support entering spouse*	--	--
Age limit	*Temp. law (2016–2018): Over 21 years applies for family reunification when reference person is a refugee allowed temp. res. under the temp. law*	--	--
Domestic violence record	Migration board checks previous marriage or partnership and, and any criminal records		
Length of residence/Residence status	Perm.res.-status required *Temp. law (2016–2018): All asylum seekers achieve only temp. res-status. Only Geneva refugees right to family reun. (if applied within 3 months)*	--	--
Attachment to country	*--*		--

## Convergence or continued difference? Family migration policy as a domain of civic integration

We began this paper by arguing that civic integration involves further aspects beside those analyzed by Sara Wallace Goodman: it is not only about language competence, historical and civic knowledge, values and loyalty oaths, though Denmark and to a lesser degree Norway certainly emphasize these traditionally civic requirements. The point that many countries are engaging in a ‘civic turn’ is just the beginning of the discussion, and we suggested that there may be a specifically Nordic version of the good citizen. Civic integration in Sweden, Denmark, and Norway is about inculcating the importance, indeed the moral requirement, of work, productivity and economic contribution to the welfare state. It is also about developing an egalitarian, autonomy-enhancing way of life, particularly in relation to gender relations and ideals of the good work life. This comprehensive (and intrusive, state-promoted) ‘liberalism’ may even be about childrearing and where and how you live. And it emerges, not least, in the area of family reunification policy and discourse.

Family reunification, interesting in its own right as a new field of civic integration, provides a useful lens through which to examine the particularities of Scandinavian civic integration policies. It also helps us investigate significant differences in national understandings of the proper means towards this civic integration (whose content and goals are more or less shared) within the region. The Scandinavian countries may stand out in the Western world, but each country implements particular policies, each with its own conception of the functionality of civic integration – with the biggest contrast between Sweden and Denmark. Besides demonstrating this geographic variation in policy and discourse, and highlighting the countries’ contrasting integration philosophies, the article’s analysis of these developments over time suggests the usefulness of distinguishing between a series of stages in the rise and possibly fall (or crowding out) of civic integration concerns in family reunification policies. This concluding discussion examines each of these points in turn. What, to begin with, characterizes family reunification as a distinct policy area?

*First*, it is directed towards both an already-resident sponsor and a (would-be) entering spouse (or child), constituting a double conditionality. It measures and adjudicates who, among the former, deserves to be allowed to (re)establish and lead a family life. And it submits the latter – in Denmark and Norway – to a regime of local integration courses and programs. The deservingness of sponsors is evaluated in employment and ability to self-support, but also, in Denmark and Norway, and for some in Sweden after recent changes, the ability to support the spouse – a paradox in universalistic welfare states traditionally hostile to family dependency. In Denmark, and more recently in Norway, it also reflects the presumed maturity and independence which is associated with age (the 24-year rule). It may further require the capacity to live an independent family life – in a suitably sized flat, away from in-laws, and even, in the former Danish point system, the potential for societal integration as signaled by not living in a “ghetto.” In Denmark, and now in Norway as well, a spill-over is also visible from the tightening of (language, waiting-period and employment) requirements for permanent residence status, which is required to have the right to import a spouse.

The integration potential of the incoming spouse is furthered in Norway and Denmark with obligatory, social benefit-conditioning language and labour-market training programs. In Denmark, these are joined with required tests, integration contracts, and a declaration on norms of active citizenship and integration that details egalitarian, non-authoritarian family life. In addition, the Danish policies include an activation-or-education regime for young people is tougher than in Norway or especially Sweden, where participation in activation plans are linked only to economic subsidies, not permanent residence or national citizenship. Also, while Denmark has further tightened its tough language and work requirements for permanent residence (for all immigrants, also family reunified), Sweden has remained committed – at least until current changes in the wake of the 2015 refugee crisis – to the idea that immediate security of residence is the best way to promote integration.

*Second*, family reunification policy also affects the “moral claims of insiders” (Block, 2012), including citizens and permanent residents who wish to marry a foreigner and the aspirations of those who, like many refugees, have only just made it inside and long for their loved ones. Historically, it has affected the former group more. While international conventions have generally shielded refugees’ right to family life (at least until recently), and EU freedom-of-movement has granted citizens of other EU countries broad rights to family reunification, citizens face nearly as many obstacles as *Gastarbeiter* descendants, whom governments some wanted to target in the first place. A class aspect and ethnic hierarchy ensues here. In Norway and Denmark pensioners, the unemployed, and even people in low income jobs cannot earn the right to bring in a spouse. Further, in Denmark some foreign spouses have been evaluated as better than others, both legally – with the most exclusionary example the former point system for high education and integration-feasible countries of origin – and in terms of discours: older men bringing in Thai wives from Thailand may more acceptable than the arranged marriages of young ethnic Turks and Pakistanis, and everybody laments the plight of the young professional who has fallen in love with an American.

A *third* feature of family reunification policy is its popular and political association, aggravated by the refugee crisis, with the spectre of an uncontrollable and multiplying inflow of migrants seen to threaten the sustainability of the welfare state and (in Denmark and Norway) the cultural cohesion of society.

Fourth, this produces a clash with those ideals of human rights, universalism and equal treatment of persons that historically have constituted core values of the welfare-era Scandinavian states. Examining the progression of policies over time, a sequence of overlapping political rationales comes into view, beginning exactly with this clash. The humanitarian emphasis on the right to family life of refugees and immigrants was the common starting point in the eighties and early nineties, but the countries’ discourses and policies have gone separate ways since then. While Danish restrictions were accompanied at an early stage with increasing marginalization of legal and human rights-based criticism, the equal right of all resident members of ‘*Folkhemmet’* (the People’s Home) remains a lodestar in Sweden, despite recent cracks in that crown. Norwegian politicians, somewhere in between, still ritually (if arguably in bad faith) insist that harsh policies respect the spirit and letter of the conventions.

The emphasis on civic integration of spouses and resident sponsors came first in Denmark in the late 1990s, spreading towards Norway, which continued to have a less conditioning version; but it never got much of a foothold in Sweden beyond a symbolic support requirement, introduced in 2010. Civic integration in Denmark, since the mid-2000s, has increasingly been complemented by civic selection, or the notion that only those individuals who have the potential to be contributing citizens and who fit the country’s liberal egalitarian way of (family) life should be allowed in. Hence the attempt to avoid entry of foreign spouses who are too young, with little education or from the wrong countries (in the former point system), as those seen as likely to live segregated lives. This type of selection, which also involves children caught by the ‘integration potential’ requirement, has come to be openly defended by Danish politicians of different stripes. Yet it was always anathema in multicultural Sweden’s discourse and policy as being completely at odds with an integration philosophy that sees every human as being willing and capable of contributing eventually. In Norway, civic selection logic may indirectly inform the very high self-support requirements on sponsors that privilege couples with high incomes and good educations, but there it has few open defenders.

If civic selection by and large remains a Danish specialty, all three countries appear to be succumbing, in various degrees and with endpoints that remain to be seen, to a controlling-the-numbers type of logic, which only indirectly bears a civic imprint, but which increasingly crowds out the politics of civic integration. Even in Sweden, the prohibitive costs of receiving more than 160 000 refugees in a single year – and the experience of doing with little willingness by EU-partners other than Germany to share the burden – has taken the country into new territory, removing the right to family reunification of non-Geneva refugees (along with all asylum seekers’ right to permanent residence). In Sweden, however, the logic of numbers is still largely seen as a matter – a very real one – of overburdened administrative processing and short-term absorptive capacity of a country that most citizens still regard as fundamentally open. In Denmark and increasingly Norway, it runs deeper, with existential fears of economic unsustainability and the future welfare-state incapacity, and decreasing ‘social cohesion’ in the wake of excessive cultural and religious pluralism.

Welfare state civic integration in the Scandinavian countries occurs within a field of significant ideological tension, particularly evident in family reunification policy, between a neo-liberal discourse about individual duty to be self-supporting and autonomous, and a more traditional social democratic ideal emphasizing the equal right and material opportunity to do so. Sweden, despite a track record of welfare state deregulation and slimming larger than Denmark’s, leans more towards the latter than its small ‘brother country’ when it comes to immigrants, as Norway adopts its traditional position in the middle. Another way to describe this contrast is to see the philosophy of integration of the three countries, in functional terms, as somewhat distinct, although the aims of integration are largely similar. In Denmark, with its 24-year-rule, contracts, declarations, and tough conditionalities, the civic integration of incoming family members is steered, engineered and indeed forced, while in Norway, with this country’s emphasis on economic self-sufficiency, it could rather be described as incentivized. In Sweden, by contrast, it remains state-facilitated.

This article has attempted to describe and compare these trajectories rather than explaining them. Even so, they fit a familiar pattern of diverse national public philosophies in otherwise similar egalitarian welfare states, laid out in other articles of this special issue (Brochmann et al., this volume). Liberal and multicultural Sweden, with its early, state-centered and internationalist conception of civic nationhood, remains very unwilling to compromise on its ideals of universalistic equal treatment, indeed unwilling to accept any conditioning of membership inclusion (Borevi, 2017; Jensen, 2016). In Denmark, the mainstream parties show few such reservations, but follow more populist and society-centered integration perceptions; the country’s tough civic integration policies still bear the culturalized imprint of its more ethno-cultural communitarian past of nineteenth-century nation building (Mouritsen, 2006 Mouritsen & Olsen, 2013). While Norway is historically closer to Denmark in terms of traditional ethno-cultural nationhood, it is also characterized by a strong humanitarian and progressive view of its place in the world and was long more inspired by Swedish multiculturalism. Yet it nevertheless now looks more to its southern neighbor, with whom it shares concerns over cultural cohesion and national identity – so inhabits an unstable middle ground that arguably makes it more sensitive to exogenous ruptures than Denmark and Sweden, which remain more set in their resilient, familiarly antagonistic ways.
